# The diagnostic and prognostic significance of HOXC13-AS and its molecular regulatory mechanism in human cancer

**DOI:** 10.3389/fmolb.2025.1540048

**Published:** 2025-02-06

**Authors:** Xiaosi Gu, Xin Hu, Sijia Zhang, Xiaoyu Zhang, Yong Wang, Lianlian Li

**Affiliations:** ^1^ Department of Immunology, School of Clinical and Basic Medical Sciences, Shandong First Medical University, Jinan, Shandong, China; ^2^ Shandong Provincial Engineering Research Center for Bacterial Oncolysis and Cell Treatment, Jinan, Shandong, China; ^3^ Laboratory of Metabolism and Gastrointestinal Tumor, The First Affiliated Hospital of Shandong First Medical University, Jinan, Shandong, China

**Keywords:** long non-coding RNA, HOXC13 antisense RNA, human tumors, biological marker, regulatory mechanisms

## Abstract

HOXC13 antisense RNA (HOXC13-AS, also known as HOXC-AS5) is a long non-coding RNA that is expressed abnormally in various types of tumors and is closely related to clinical staging, clinical pathological features, and patient survival. HOXC13-AS is involved in the occurrence and development of tumors, affecting cell proliferation, migration, invasion, epithelial–mesenchymal transition, and tumor growth. This review summarizes the clinical significance of HOXC13-AS as a biomarker for human tumor diagnosis and prognosis and outlines the function and molecular regulation mechanism of HOXC13-AS in various types of cancer, including nasopharyngeal carcinoma, breast cancer, oral squamous cell carcinoma, glioma, and cervical cancer. Overall, this review emphasizes the potential of HOXC13-AS as a human tumor predictive biomarker and therapeutic target, paving the way for its clinical application.

## 1 Introduction

For decades, cancer has been the leading cause of human health and death. According to the latest statistical data, there were nearly 20 million new cancer cases and 9.7 million cancer-related deaths worldwide in 2022 (Bray et al., 2024). It is predicted that by 2050, the number of new cancer cases will reach 35 million (Bray et al., 2024). In 2022, there were approximately 4.82 million new cancer cases and 2.57 million new cancer-related deaths in China (Han et al., 2024). In the United States, it is expected that there will be approximately 2 million new cancer cases and 0.61 million cancer-related deaths by 2024 (Siegel et al., 2024). These data reflect that unremitting efforts in cancer prevention, diagnosis, and treatment are required to reduce the burden of cancer.

During this period, it was discovered that long non-coding RNAs (lncRNAs) in regulatory non-coding RNAs can extensively participate in the occurrence and development of cancer at various levels (Rajakumar et al., 2023) and can be developed into diagnostic, therapeutic, and prognostic biomarkers (Chi et al., 2019; Goyal et al., 2021; Najafi et al., 2022; Zhang et al., 2023). LncRNA is a type of non-coding RNA with length exceeding 200 nucleotides (Pierouli et al., 2022). LncRNA is believed to play a role in many cellular processes, including cell cycle (Hashemi et al., 2022; Yin et al., 2023; Zangouei et al., 2023), differentiation (Liu et al., 2022; Wang and Qi, 2021), apoptosis (Iwai et al., 2023; Wang et al., 2023), migration (Ye et al., 2021), and metabolism (Liu et al., 2021; Tan et al., 2021a).

**FIGURE 1:**
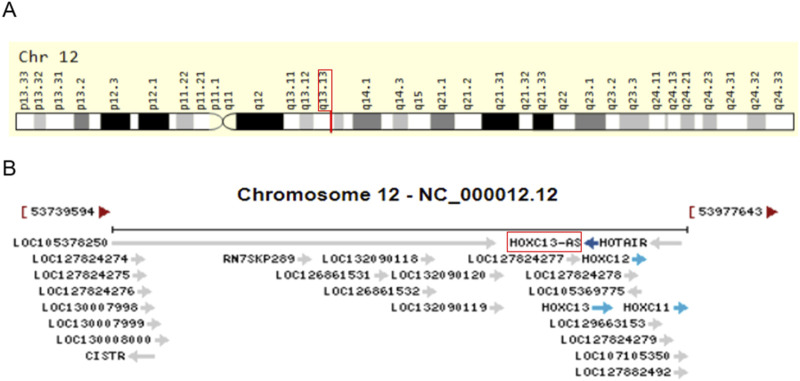
Genomic view for HOXC13-AS gene for genomic location **(A)** extracted from GeneCards database (https://www.genecards.org/cgi-bin/carddisp.pl?gene=HOXC13-AS&keywords=HOXC13-AS), and genomic context **(B)** from NCBI database (https://www.ncbi.nlm.nih.gov/gene/100874366).

The HOX gene is a member of the homeobox superfamily, and its gene sequence is highly conserved (Li et al., 2019a). There are 39 HOX genes in humans, divided into four clusters: HOXA, HOXB, HOXC, and HOXD. The HOX cluster also contains many non-coding RNAs, including some lncRNAs. The expression of these lncRNAs has exhibited abnormalities in different types of cancer and is associated with the occurrence and progression of cancer, such as HOXA cluster antisense RNA2 (Chen and He, 2021; Jiang et al., 2019; Wang et al., 2019; Zhang et al., 2022a; Zheng et al., 2019), HOXB cluster antisense RNA3 (Jiang et al., 2020; Wu et al., 2024; Xing et al., 2021a; Xu et al., 2021), and HOXC cluster antisense RNA3 (Su et al., 2022; Su et al., 2020; Yang et al., 2021; Zhang et al., 2022b; Zhao et al., 2021). In addition to the aforementioned lncRNAs, HOXC13 antisense RNA (HOXC13-AS, also known as HOXC-AS5) has attracted attention due to its abnormal expression patterns found in many malignant tumors. HOXC13-AS is an antisense lncRNA transcribed from chromosome 12. It is located on the reverse strand of chromosome 12 (Figure 1A) (https://www.genecards.org/cgi-bin/carddisp.pl?gene=HOXC13-AS&keywords=HOXC13-AS) (Stelzer et al., 2016), with coordinates 53935328–53939643, spans three exons, and has a length of 4,316 nucleotides (source https://www.ncbi.nlm.nih.gov/gene/100874366). In addition, HOXC13-AS overlaps with the HOXC13 gene on the sense strands. More interestingly, it shares genomic position 12q13.13 with HOX transcript antisense RNA (Figure 1B) [National Center for Biotechnology Information (NCBI) (Internet). Bethesda (MD): National Library of Medicine (US), National Center for Biotechnology Information (1988)–(cited 2025 January 10). Available from: https://www.ncbi.nlm.nih.gov/]. The oncogenic lncRNA HOX transcript antisense RNA is involved in the occurrence and development of various types of cancer (Fu et al., 2016; Guo et al., 2023; Shi et al., 2022; Wei et al., 2020; Zhang et al., 2024), indicating the potential role of HOXC13-AS in cancer. Some studies have found that the expression of HOXC13-AS is dysregulated in various types of malignant tumors, and upregulated HOXC13-AS expression is associated with clinical pathological features such as tumor node metastasis (TNM) staging, metastasis, clinical staging, and prognosis (Gao et al., 2019; Zhou J. F. et al., 2019). More studies have found that HOXC13-AS is involved in the occurrence and development of tumors by affecting processes such as cell proliferation, invasion, migration, and epithelial–mesenchymal transition (EMT) (Gao et al., 2019; Li et al., 2020; Li et al., 2019b; Liu et al., 2019; Wang et al., 2021). HOXC13-AS has exhibited potential application value as a tumor treatment, a prognostic biomarker, and a therapeutic target.

In this review, we systematically searched PubMed and Web of Science databases using the keywords “HOXC13-AS,” “HOXC13 antisense RNA,” and “HOXC-AS5” and screened for research literature related to human cancer. Based on the aforementioned search results, this review summarizes the expression levels of HOXC13-AS in different types of cancer and its correlation with clinical pathological characteristics, prognosis, and diagnostic value. This review also outlines the biological role and molecular regulatory mechanisms of HOXC13-AS in various types of cancer, aiming to lay a foundation for the clinical application of HOXC13-AS.

## 2 Expression of HOXC13-AS and its clinical significance as a potential biomarker in human cancer

Many studies have revealed the abnormal expression of HOXC13-AS in various types of human tumors, and it has been established that the dysregulation of HOXC13-AS is associated with certain clinical pathological characteristics and the prognosis of patients (Table 1). This section provides an overview of the expression changes of HOXC13-AS in human malignancies and its correlation with some clinical pathological characteristics and discusses the potential of HOXC13-AS as a valuable biomarker for prognosis in different types of tumors.

**TABLE 1:** Expression of HOXC13-AS in tissue samples and its relationship with clinical characteristics and survival in human tumors^a^.

Cancer type	Expression level	Model used	Clinical characteristics	Survival indicator	Prognosis with high expression	Biomarker	Ref.
Breast cancer	Up	Human tissues (100 paired cancer and adjacent tissues)	-	-	-	-	Li et al. (2019a)
Cervical cancer	Up	Human tissues (52 paired cancer and adjacent tissues)	-	-	-	-	Wang et al. (2021)
Glioma	Up	Human sample (20 cancer and 7 non-cancerous tissues), TCGA	-	Overall survival (*p* < 0.001)	Adverse	Prognostic	Liu et al. (2019)
Hepatocellular carcinoma	Up	Human tissues (197 paired cancer and adjacent tissues)	TNM stage, lymph node metastasis	Overall survival (*p* < 0.0106), disease-free survival (*p* < 0.0066)	Adverse	Prognostic	Zhou J. F. et al. (2019)
Head and neck squamous cell carcinoma	Up	TCGA (141 tumor,44 normal)	-	-	-	Diagnostic	Xiong et al. (2020)
Intrahepatic cholangiocarcinoma	Up	Human sample (39 tumor samples)	-	Overall survival (*p* = 0.047), disease-free survival (*p* = 0.048)	Adverse	Prognostic	Angenard et al. (2019)
Nasopharyngeal carcinoma	Up	Human tissues (84 paired cancer and normal tissues)	Local-regional recurrence, distant metastasis, clinical stage	Overall survival (*p* = 0.010)	Adverse	Prognostic	Gao et al. (2019)
Oral squamous cell carcinoma	Up	Human tissues (56 pairs of cancer and non-cancerous tissues)	-	-	-	-	Li et al. (2020)

^a^ TNM, tumor node metastasis; HOXC13-AS, HOXC13 antisense RNA, TCGA, the cancer genome atlas.

### 2.1 Expression of HOXC13-AS in human cancer

Previous studies have revealed that HOXC13-AS is abnormally expressed in various types of human cancer, including hepatocellular carcinoma, nasopharyngeal carcinoma, breast cancer, head and neck squamous cell carcinoma (HNSCC), oral squamous cell carcinoma, glioma, cervical cancer, and intrahepatic cholangiocarcinoma (Table 1).

**FIGURE 2:**
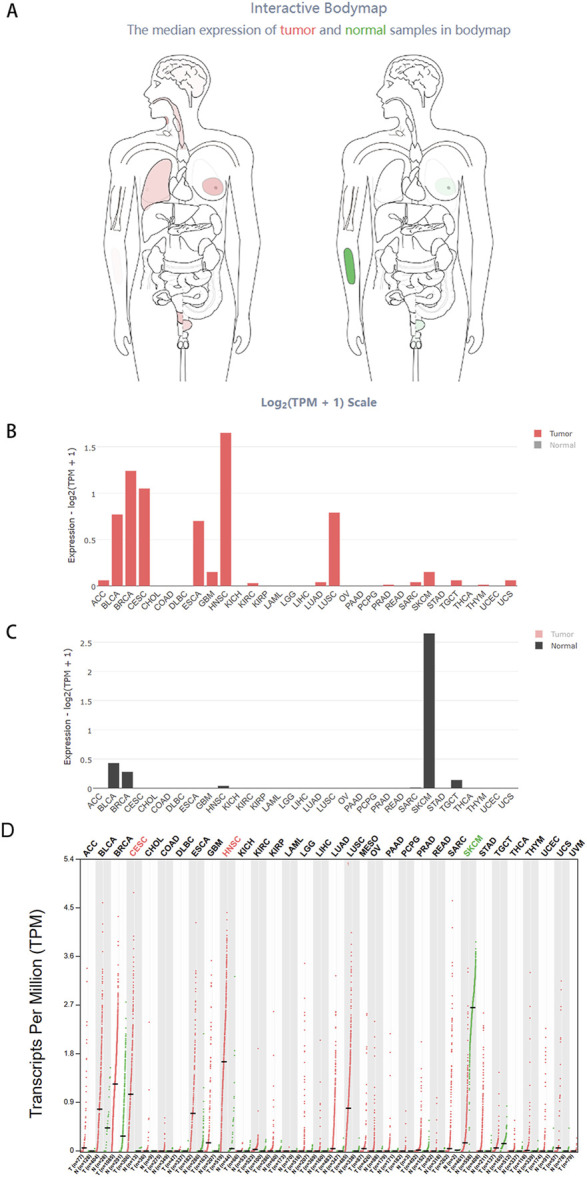
The comprehensive expression profile of HOXC13-AS in human tissues. Using data from GEPIA 2 (https://www.kmplot.com/analysis/): Interactive Bodymap, the analysis of HOXC13-AS gene expression including all identified isoforms depicted the median expression of HOXC13-AS in tumor and normal samples **(A)**. The bar chart provides detailed gene expression profiles of tumor samples **(B)** and corresponding normal tissues **(C)**. The dot plot represents the gene expression profiles of all tumor samples and paired normal tissues **(D)**. ACC, adrenocortical carcinoma; BLCA, bladder urothelial carcinoma; BRCA, breast invasive carcinoma; CESC, cervical squamous cell carcinoma and endocervical adenocarcinoma; CHOL, cholangiocarcinoma; COAD, colon adenocarcinoma; DLBC, diffuse large B-cell lymphoma; ESCA, esophageal carcinoma; GBM, glioblastoma multiforme; HNSC, head and neck squamous cell carcinoma; KICH, kidney chromophobe; KIRC, kidney renal clear cell carcinoma; KIRP, kidney renal papillary cell carcinoma; LAML, acute myeloid leukemia; LGG, brain lower grade glioma; LIHC, liver hepatocellular carcinoma; LUAD, lung adenocarcinoma; LUSC, lung squamous cell carcinoma; MESO, mesothelioma; OV, ovarian serous cystadenocarcinoma; PAAD, pancreatic adenocarcinoma; PCPG, pheochromocytoma and paraganglioma; PRAD, prostate adenocarcinoma; READ, rectum adenocarcinoma; SARC, sarcoma; SKCM, skin cutaneous melanoma; STAD, stomach adenocarcinoma; TGCT, testicular germ cell tumors; THCA, thyroid carcinoma; THYM, thymoma; UCEC, uterine corpus endometrial carcinoma; UCS, uterine carcinosarcoma; UVM, uveal melanoma.

We extensively investigated the expression of HOXC13-AS in pan-cancer. The interactive BodyMap in GEPIA2 (http://gepia2.cancer-pku.cn/) (Tang et al., 2019) identified the expression profiles of HOXC13-AS RNA transcripts in both tumor and normal tissues. According to the interactive BodyMap, HOXC13-AS is expressed at higher levels in tumor tissues than in normal tissues, primarily in malignancies of the head and neck, lung, breast, cervix uteri, and bladder (Figure 2A). In tumor samples, HNSCC exhibited the highest expression of RNA transcripts (Figure 2B), whereas in normal tissues, the highest expression was observed in normal tissues adjacent to skin cutaneous melanoma (Figure 2C). Furthermore, the results demonstrated that HOXC13-AS was significantly upregulated in many types of malignancies, including HNSCC, cervical squamous cell carcinoma, and endocervical adenocarcinoma. Conversely, skin cutaneous melanoma exhibited a marked downregulation of HOXC13-AS (Figure 2D).

### 2.2 HOXC13-AS as a prognostic and diagnostic biomarker

HOXC13-AS, as a potential biomarker in human tumors, has been reported in many studies for its enormous clinical potential (Table 1). A study has found that the expression level of HOXC13-AS is correlated with the clinical characteristics of some types of cancer (Table 1). For example, in hepatocellular carcinoma (Zhou Q. et al., 2019), high expression of HOXC13-AS is closely associated with TNM staging and lymph node metastasis. In nasopharyngeal carcinoma (Gao et al., 2019), overexpression of HOXC13-AS is significantly correlated with local recurrence, distant metastasis, and clinical staging. However, the relationship between the expression of HOXC13-AS and patient prognosis was also discovered through research. Upregulation of the expression of HOXC13-AS in hepatocellular carcinoma (Zhou J. F. et al., 2019), nasopharyngeal carcinoma (Gao et al., 2019), glioma (Liu et al., 2019), and intrahepatic cholangiocarcinoma (Angenard et al., 2019) indicates poor prognosis for patients. In addition, HOXC13-AS is a new candidate diagnostic biomarker for patients with HNSCC (Xiong et al., 2020).

**FIGURE 3:**
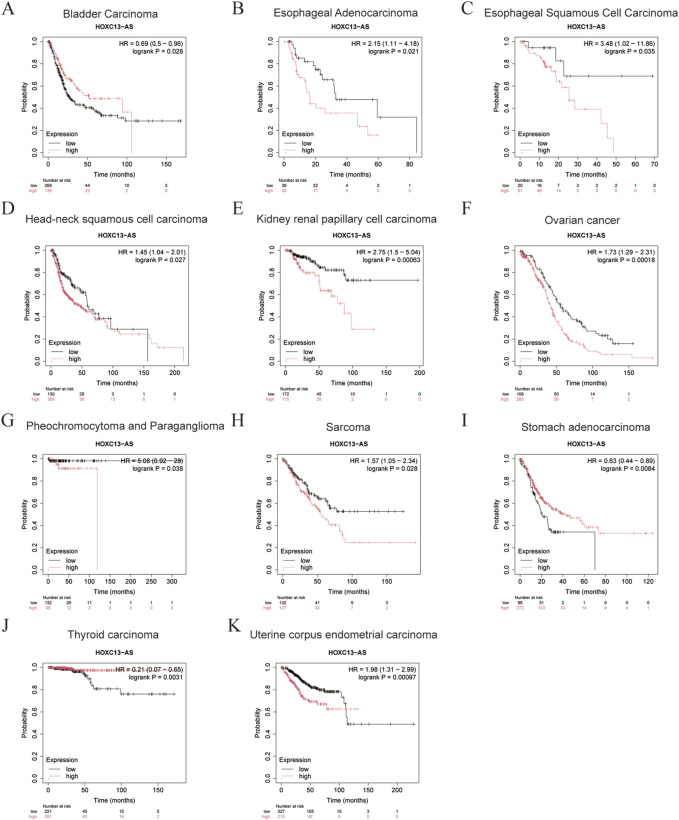
The relationship between HOXC13-AS expression and overall survival (OS) in bladder carcinoma **(A)**, esophageal adenocarcinoma **(B)**, esophageal squamous cell carcinoma **(C)**, head-neck squamous cell carcinoma **(D)**, kidney renal papillary cell carcinoma **(E)**, ovarian cancer **(F)**, pheochromocytoma and paraganglioma **(G)**, sarcoma **(H)**, stomach adenocarcinoma **(I)**, thyroid carcinoma **(J)**, uterine corpus endometrial carcinoma **(K)** from the Kaplan–Meier plots (https://www.kmplot.com/analysis/). HOXC13-AS: HOXC13 antisense RNA; HR: hazard rate.

We analyzed the relationship between HOXC13-AS expression levels and prognosis using the Kaplan-Meier plots (https://www.kmplot.com/analysis/) (Győrffy, 2024). The expression level of HOXC13-AS is significantly related to the prognosis of patients with bladder cancer, esophageal adenocarcinoma, esophageal squamous cell carcinoma, HNSCC, kidney renal papillary cell carcinoma, ovarian cancer, pheochromocytoma and paraganglioma, sarcoma, stomach adenocarcinoma, thyroid carcinoma, and uterine corpus endometrial carcinoma (Figure 3). The high expression of HOXC13-AS indicates poorer overall survival in esophageal adenocarcinoma, esophageal squamous cell carcinoma, kidney renal papillary cell carcinoma, ovarian cancer, pheochromocytoma and paraganglioma, and sarcoma (Figures 3B, C, E–H), whereas its high expression indicates that overall survival is better in stomach adenocarcinoma and thyroid carcinoma, which can serve as favorable predictive factors (Figures 3I, J). These findings indicate that HOXC13-AS has different prognostic significance in various types of cancer, rendering it a potentially effective prognostic biomarker for a range of human malignancies.

## 3 The molecular mechanisms and role of HOXC13-AS in cancer

**FIGURE 4:**
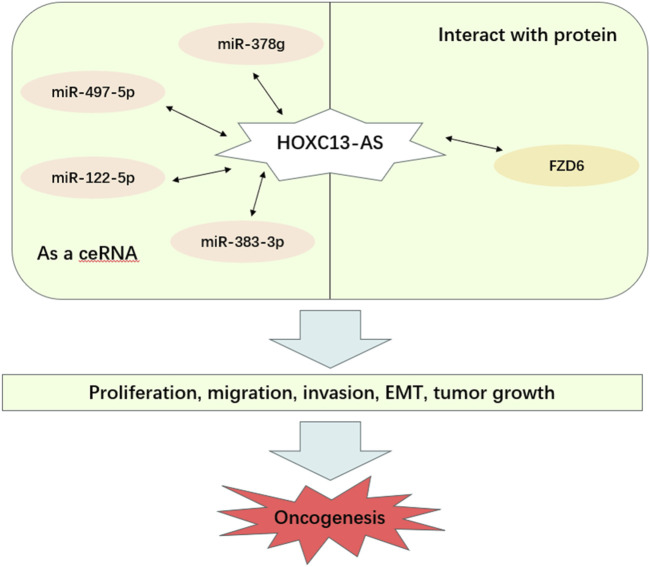
Main mechanisms of HOXC13-AS in cancer progression. HOXC13-AS, HOXC13 antisense RNA; FZD6, frizzled class receptor 6; ceRNA, competing endogenous RNA; EMT, epithelial mesenchymal transition.

**FIGURE 5:**
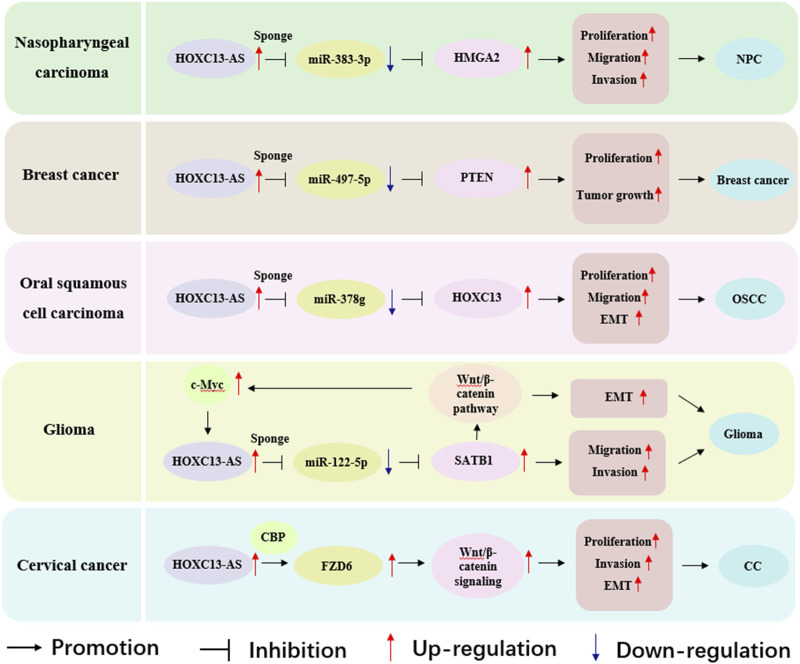
Role of HOXC13-AS in modulating molecular pathways across nasopharyngeal carcinoma, breast cancer, oral squamous cell carcinoma, glioma and cervical cancer. HOXC13-AS, HOXC13 antisense RNA; HMGA2, high mobility group AT-hook 2; NPC, nasopharyngeal carcinoma; PTEN, phosphatase and tensin homolog; EMT, epithelial mesenchymal transition; OSCC, oral squamous cell carcinoma; SATB1, special AT rich sequence binding protein 1; CBP, cAMP-response element binding protein-binding protein; FZD6, frizzled class receptor 6; CC, cervical cancer.

HOXC13-AS participates in regulating biological functions through different molecular mechanisms in various types of cancer, thereby affecting the occurrence and development of cancer. These biological functions include cell proliferation, migration, invasion, EMT, and tumor growth (Table 2; Figure 4). Based on these findings, HOXC13-AS is expected to become a new target for human cancer treatment. Many studies have explored the regulatory mechanism of lncRNA HOXC13-AS in different types of cancer (Figure 5), including nasopharyngeal carcinoma (Gao et al., 2019), breast cancer (Li et al., 2019a), oral squamous cell carcinoma (Li et al., 2020), glioma (Liu et al., 2019), and cervical cancer (Wang et al., 2021). Among these regulatory mechanisms, the most common one is HOXC13-AS as a competing endogenous RNA (ceRNA) to bind with microRNA (miRNA). LncRNAs can be seen as a “sponge” to competitively bind miRNA through their specific sequence, which are complementary to the miRNA, thus reducing the regulatory effects of miRNA to downstream target genes (Ma et al., 2023). In this section, the molecular mechanisms underlying the regulatory role of HOXC13-AS in different types of malignant tumors are discussed.

**TABLE 2:** The role and regulatory mechanism of HOXC13-AS in human malignancies^a^.

Cancer type	Role	Experiment	Functions	Mechanism of action of HOXC-AS3	Related molecule/signal	Sponging miRNA sequence (3′-5′)	Ref.
Breast cancer	Oncogenic	*In vitro* and *in vivo*	Proliferation, tumor growth	As a ceRNA	MiR-497-5p, PTEN	UGUCACAC	Li et al. (2019b)
Cervical cancer	Oncogenic	*In vitro*	Proliferation, invasion, EMT	Interact with protein	FZD6, Wnt/β-catenin signaling pathway	-	Wang et al. (2021)
Glioma	Oncogenic	*In vitro*	Migration, invasion, EMT	As a ceRNA	MiR-122-5p, SATB1, c-Myc, Wnt/β-catenin signaling pathway	UGUGAGGU	Liu et al. (2019)
Hepatocellular carcinoma	Oncogenic	*In vitro*	-	-	-	-	Zhou J. H. et al., (2019)
Nasopharyngeal carcinoma	Oncogenic	*In vitro*	Proliferation, migration, invasion	As a ceRNA	MiR-383-3p, HMGA2	AGACUGCUCC、GUCACGAC	Gao et al. (2019)
Oral squamous cell carcinoma	Oncogenic	*In vitro* and *in vivo*	Proliferation, migration, EMT	As a ceRNA	MiR-378g, HOXC13	UCGGGUCA	Li et al. (2020)

^a^ EMT, epithelial–mesenchymal transition; ceRNA, competing endogenous RNA; PTEN, phosphatase and tensin homolog; FZD6, frizzled class receptor 6; SATB1, special AT-rich sequence binding protein 1; HMGA2, high mobility group AT-hook 2; HOXC13-AS, HOXC13 antisense RNA.

### 3.1 Nasopharyngeal carcinoma

Nasopharyngeal carcinoma (NPC) is a type of HNSCC that predominantly occurs in East Asia and Southeast Asia (Chen et al., 2019). The expression of HOXC13-AS was observed to increase in NPC tissues, and HOXC13-AS was highly expressed in five cancer cell lines (CNE 1, 6-10B, SUNE 2, HNE-1, and CNE 2), exhibiting a significant correlation with local-regional recurrence, distant metastasis, and clinical stage (Gao et al., 2019). In addition, high expression of HOXC13-AS is associated with poor patient prognosis and low overall survival (Gao et al., 2019). Functionally, *in vitro* experiments have indicated that knocking down HOXC13-AS inhibits cell proliferation, migration, and invasion (Gao et al., 2019). Mechanistically, HOXC13-AS acts through the HOXC13-AS–miR-383-3p–high mobility group AT-hook 2 (HMGA2) axis, where HOXC13-AS functions as a ceRNA and promotes NPC progression by competitively sponging with miR-383-3p to enhance the expression of HMGA2 (Gao et al., 2019). In summary, although HOXC13-AS exerts oncogenic function by regulating the miR-383-3p/HMGA2 axis, it also provides new ideas for the treatment of nasopharyngeal carcinoma patients to a certain extent.

### 3.2 Breast cancer

Breast cancer is one of the most common malignant tumors in women and is also a major cause of cancer-related deaths in women worldwide (Wilkinson and Gathani, 2022). Real-time quantitative polymerase chain reaction, Cell Counting Kit-8 assay, and colony formation assay demonstrated that compared with adjacent normal tissues, the expression of HOXC13-AS in breast cancer tissues was significantly upregulated, and the upregulated HOXC13-AS promoted the growth of breast cancer cells (Li et al., 2019b). In the mechanism study, *in vivo* and *in vitro* experiments demonstrated that HOXC13-AS promotes breast cancer cell proliferation and tumor growth through the miR-497-5p/phosphatase and tensin homolog (PTEN) axis. Put differently, HOXC13-AS, which is significantly upregulated in breast cancer, can act as the “sponge” of miR-497-5p, reduce the expression of miR-497-5p, and further increase PTEN to promote cell proliferation (Li et al., 2019a). These findings suggest that HOXC13-AS may play a carcinogenic role in breast cancer, but it is expected to become a potential target for treatment.

### 3.3 Oral squamous cell carcinoma

Studies have found that HOXC13-AS is upregulated in oral squamous cell carcinoma (OSCC) tissues and cell lines, leading to oncogenic functions (Li et al., 2020). HOXC13-AS upregulates the expression of HOXC13 in OSCC cells by sequestering miR-378g, promoting OSCC cell proliferation, migration, and EMT (Li et al., 2020). The expression of HOXC13 is positively correlated with HOXC13-AS and negatively correlated with miR-378g. Overall, HOXC13-AS, as a ceRNA, promotes OSCC development through the HOXC13-AS/miR-378g/HOXC13 axis (Li et al., 2020), providing a new approach for lncRNA targeted therapy of OSCC.

### 3.4 Glioma

Gliomas account for approximately 80% of all intracranial malignancies (Li et al., 2022). HOXC13-AS is considered an oncogene of glioma (Liu et al., 2019). The expression of HOXC13-AS is elevated in the tissues and cells of gliomas, and high levels of HOXC13-AS indicate poor prognosis in patients. Knockdown of HOXC13-AS can hinder the migration, invasion, and EMT process of glioma cells (Liu et al., 2019). Mechanistically, HOXC13-AS as a ceRNA indirectly regulates special AT-rich sequence binding protein 1 (SATB1) expression by sponging miR-122-5p, thereby affecting the EMT process, and the Wnt/β-catenin pathway is also involved in this process (Liu et al., 2019). Interestingly, the Wnt/β-catenin pathway target gene c-Myc can regulate the expression of HOXC13-AS at the transcriptional level by binding to the promoter region of HOXC13-AS, thereby forming a positive feedback loop (Liu et al., 2019). The discovery of the HOXC13-AS–miR-122-5p–SATB1–c-Myc feedback loop has improved the regulatory mechanism of HOXC13-AS in malignant tumors and provided potential therapeutic targets for gliomas (Liu et al., 2019).

### 3.5 Cervical cancer

Cervical cancer (CC) is the fourth most common cancer among women worldwide and the leading cause of cancer-related deaths among women in developing countries (Buskwofie et al., 2020). HOXC13-AS is highly expressed in CC tissues and cell lines and is positively correlated with frizzled class receptor 6 (FZD6) (Wang et al., 2021). HOXC13-AS upregulates FZD6 and activates Wnt/β-catenin signaling to promote CC proliferation, invasion, and EMT (Wang et al., 2021). HOXC13-AS upregulates FZD6 through interaction with cAMP-response element binding protein-binding protein (CBP), inducing histone H3 lysine 27 acetylation on the FZD6 promoter (Wang et al., 2021). Fat mass and obesity-associated protein improves the stability of HOXC13-AS by reducing *N* ^6^-methyladenosine (Wang et al., 2021). In general, FZD6 is an oncogene in CC, and HOXC13-AS has great potential as a new target for CC therapy.

## 4 Conclusions and perspectives

Numerous lines of evidence have indicated that multiple lncRNAs are involved in the occurrence and development of human tumors through various complex mechanisms (Chen et al., 2022; Hashemi et al., 2022; McCabe and Rasmussen, 2021; Xing et al., 2021b), and HOXC13-AS is one of them, playing an important role in tumor research. Studies have found that HOXC13-AS is dysregulated in various types of human tumors (Angenard et al., 2019; Gao et al., 2019; Li et al., 2020; Li et al., 2019b; Liu et al., 2019; Wang et al., 2021; Xiong et al., 2020; Zhou Q. et al., 2019). The expression level of lncRNA HOXC13-AS is closely related to the clinical characteristics of tumors. For example, in hepatocellular carcinoma, HOXC13-AS levels are significantly correlated with TNM staging and lymph node metastasis, and high expression levels of HOXC13-AS indicate advanced liver cancer status in patients (Zhou J. F. et al., 2019). In nasopharyngeal carcinoma, the expression level of HOXC13-AS is correlated with local recurrence, distant metastasis, and clinical staging, further demonstrating the effect of HOXC13-AS on cancer-related features (Gao et al., 2019). Moreover, HOXC13-AS is involved in various biological processes. High expression of HOXC13-AS promotes cell proliferation, migration, invasion, EMT, and tumor growth, thereby accelerating tumor progression (Gao et al., 2019; Li et al., 2020; Li et al., 2019a; Liu et al., 2019; Wang et al., 2021).

HOXC13-AS has the potential to become a prognostic and diagnostic biomarker. It presents unique prognostic factors in different types of cancer and provides new ideas and insights for cancer treatment. According to relevant research reports, in nasopharyngeal carcinoma and glioma, patients with higher levels of HOXC13-AS have a lower overall survival rate than those with lower levels of HOXC13-AS (Gao et al., 2019; Liu et al., 2019). Similarly, in hepatocellular carcinoma and intrahepatic cholangiocarcinoma, the expression level of HOXC13-AS is negatively correlated with overall survival and disease-free survival (Angenard et al., 2019; Zhou Q. et al., 2019). In addition, HOXC13-AS is a new candidate diagnostic biomarker for patients with HNSCC (Xiong et al., 2020). Furthermore, it was found that the high expression of HOXC13-AS in different cancers represents different prognostic effects though a comprehensive analysis of Kaplan-Meier plots (Figure 3). It indicated poor prognosis in esophageal adenocarcinoma, esophageal squamous cell carcinoma, kidney renal papillary cell carcinoma, ovarian cancer, pheochromocytoma and paraganglioma, and sarcoma, while it indicated good prognosis in bladder carcinoma, stomach adenocarcinoma and thyroid carcinoma. This variability in prognosis might be caused by tissue-specific expression of lncRNA (Galamb et al., 2019; Sarropoulos et al., 2019).

The functions of lncRNA are diverse (Nemeth et al., 2024). It can directly interact with DNA (Arab et al., 2019; Luo et al., 2022), RNA (Hu et al., 2014; Shi et al., 2020; Tan et al., 2021b), and also proteins (Ferrè et al., 2016; Li et al., 2021; Zhang et al., 2018; Zhou J. F et al., 2019). In some types of cancer, the molecular mechanism of HOXC13-AS is achieved by interacting with microRNAs to exhibit sponge-like activity, which in turn regulates the expression of target genes to exert its regulatory effect. For example, in nasopharyngeal carcinoma, HOXC13-AS sponges miR-497-5p, forming a ceRNA network to enhance the expression of HMGA2, which means that it participates in tumor progression through the HOXC13-AS–miR-383-3p–HMGA2 axis (Gao et al., 2019). Similarly, in breast cancer, HOXC13-AS plays a role through the miR-497-5p/PTEN axis (Li et al., 2019b). In oral squamous cell carcinoma, HOXC13-AS affects tumor development through the miR-378g/HOXC13 axis (Li et al., 2020). In gliomas, HOXC13-AS exerts regulatory effects through the HOXC13-AS–miR-122-5p–SATB1–c-Myc feedback loop (Liu et al., 2019). In addition, HOXC13-AS can interact with proteins along with microRNAs. In cervical cancer, HOXC13-AS upregulates FZD6 by interacting with CBP, thereby activating Wnt/β-catenin signaling to accelerate tumor progression (Wang et al., 2021). In glioma, nasopharyngeal carcinoma, breast cancer and oral squamous cell carcinoma, HOXC13-AS is expected to become a potential target for treatment. Interestingly, HOXC13-AS plays a role in these tumors through the lncRNA-miRNA axis. And this axis is particularly important in clinical trials, as the two non-coding RNA molecules in the axis can regulate the effects of drugs on the body, reduce cell resistance, and provide new ideas for the development and application of targeted new drugs (Entezari et al., 2022; Ma et al., 2023). The discovery of these molecular mechanisms suggests that HOXC13-AS may become a potential target for cancer therapy, enhancing the sensitivity of cancer treatment.

Although there has been progress in the study of the role of HOXC13-AS in human tumors, there are still several issues that remain to be explored and resolved. Firstly, Figures 2B–D shows that HOXC13-AS is highly expressed in various cancers, but there is a lack of research on the upstream regulatory processes of HOXC13-AS. Existing research suggests that upregulation of lncRNA genes may be related to transcription factor activation (Wei et al., 2016), N6-methyladenosine demethylation (Yao et al., 2024), single nucleotide polymorphism (Yan et al., 2021), and other factors. Therefore, further exploration of the upstream mechanisms of HOXC13-AS is still needed. Secondly, there is a lack of existing research on the molecular mechanisms and related signaling pathways involved in the regulation of HOXC13-AS for different types of cancer, and a more comprehensive investigation is required. Therefore, there is an urgent need to search for downstream small molecules that can be paired with it to explore the corresponding molecular mechanisms and provide new targets and ideas for cancer treatment to meet the needs of developing and optimizing new treatment methods. Thirdly, more *in vivo* and *in vitro* experiments will be needed to validate the potential clinical application value of HOXC13-AS as a diagnostic and prognostic biomarker. It is expected to establish a multi–biomarker combined diagnostic or prognostic model in the near future, combining HOXC13-AS with other related biomarkers, comprehensively analyzing various indicators, and providing stronger evidence for clinical applications. During this process, due to the main regulatory role of lncRNA in the organism, its copy number is low and its expression is restricted in different developmental stages and cells, making detection difficult (Mattick et al., 2023). What’s even more complex is that most lncRNAs have different subtypes and their localization in cells varies, even if they are located the same, they still have different functions (Bridges et al., 2021). However, in current research on HOXC13-AS, there is a lack of studies on its subcellular localization, which is crucial for studying the function of HOXC13-AS. Therefore, further in-depth exploration is needed in this area.

In summary, HOXC13-AS plays a unique role in regulating tumor progression. A detailed and comprehensive understanding of the role of HOXC13-AS will facilitate the exploration of its clinical value as a prognostic and diagnostic biomarker and enhance its potential as a new therapeutic target. More in-depth scientific research on the diverse mechanisms of HOXC13-AS will be needed to conducted in the near future. Its plasticity in treatment resistance and drug development were looked forward to further clarify based on multi–center research cooperation, in order to promote the clinical application value of HOXC13-AS.
